# The Hospitals Association and Patients' Contributions

**Published:** 1888-09-22

**Authors:** Henry C. Burdett


### THE HOSPITALS ASSOCIATION AND PATIENTS' CONTRIBUTIONS.

SPEECH BY MR. HENRY C. BURDETT.

I SHOULD like to commence my remarks by calling attention to the present position of the hospital system in London. Briefly, it is this. We have our voluntary hospitals in the position of requiring every year £100,000 more than they actually receive from reserve—that is, revenue derived from all charitable sources and all invested property. Now, how is it, considering that this is a chronic state of affairs, that these hospitals live at all? It is simply that legacies produce a sum which is equivalent in three years to two years' deficiency. That is to say, that every third year in London there is a deficiency of £300,000, which represents the deficiency of income compared with the necessary expenditure of the London hospitals; and on the average of years the legacies produce every three years £200,000, which leaves £100,000 to be made up some way or another. That is £33,000 a year; and that £33,000 has gone on steadily increasing. It has remained during the last seven years almost stationary, entirely owing to the fact that of £500,000, the expenses of the London hospitals, £44,000 is now derived entirely from patients' payments. This is a statement of fact which ought to be looked at. I wish, however, to pass to the next point. Sir Sydney Waterlow, than whom no one has devoted more time or energy to the interests of hospitals, has defended the old system. There is no one to whom the authorities of hospitals owe a deeper debt of gratitude than to Sir Sydney, for he it is, you must remember, who has introduced Hospital Sunday in London, and has got £40,000 a-year for the hospitals. Sir Sydney Waterlow very rightly dwelt upon the fact that certain hospitals are not in a position to accept the pay system at all. That position everyone who understands the question will accept at once. These hospitals are of two kinds. They are endowed hospitals, the income of which is insufficient to maintain all the beds occupied by poor people; or they are, on the other hand, hospitals like the London Hospital, which is situated in a district where the present necessities of the population upon the number of beds provided is so urgent and so unceasing that you cannot make room for a single inch of ground even to put in another bed. It is obviously impossible to treat any patients there but the deserving poor, and I would point out, as illustrating this, that the authorities of the London Hospital were years ago impressed with the soundness, under certain conditions, of the Pay System, for they took the trouble to go to legislation in Parliament, and to get a clause incorporated in their new Act, empowering them to take paying patients. And when the first system of pay hospitals was originally seriously advocated in 1876, after two years' discussion, the managers of the London Hospital communicated with those who had raised a sum of money to support the Pay System proper in this country, to see whether it would be possible to have a paying ward in the London Hospital. Therefore, I think it is only fair to our greatest hospital that it should be plainly understood that, whereas in spirit they are in favour of the Pay System, the necessities of the poor are such that it is not a practical question with them, for the simple reason that they have not room to put beds, and positively have not room enough to put the suffering poor. With that proviso and these dissensions, I say it would be proper on the part of the managers of London hospitals to look this question of pay hospitals in the face, and to say whether it is possible or not to occupy the something like 4,000 beds which are at present available in a practical manner by the introduction of some branch of the Pay System. This is a question of the greatest importance, because any hospital which attempts to try the Pay System will find, as the American hospitals have found, that it will gradually alter the admission of the patients, it will gradually alter the tone of the patients treated, and it will produce results within the institutions which are full of good, by awakening the conscience of everybody who receives relief in that institution to a sense of his or her individual duty. That will result, as it has resulted in America, in making the men and women feel that it is an honour and an ennobling quality to do the best possible thing, even if it is only in the form of subscribing one penny, for the institution which ministers to them when they are down, and cannot work to sustain their family, so that they can say to themselves: "I have done my duty, I have done my best, and I intend to do my best for those who have enabled me to more than live—to recover the means by which I earn my living and that of my family." Then, while I attach great importance to an effort being made to fill the vacant beds in London by some adoption of the Pay System, I would say this, that there is another class of hospitals in which the adoption of the Pay System is exceedingly important, and that is in the new hospitals which must necessarily be built, and which are being built near London—for instance, the hospital of East and West Ham—in order to meet the growth of the population of London. In East and West Ham they have started a hospital which is simply promoted by the working classes, the money for which has been largely given by the working classes, and where, I hope and believe and understand that the system which has been alluded to by Dr. Jamison, and which has been brought out so exhaustively in Mr. Burdett-

Coutts's paper—in which is provided instances in all parts of the country to show that people of the working classes will take their hospitals over, and contribute *pro rata* to the expenses—will be adopted. Not only is the system a non-compulsive one, but at the North Staffordshire Infirmary they have come into this proud position, that not only do the working classes pay the whole cost of the treatment of every single person whom they send in—themselves, their wives, or their children—but they give every year a sum of £500 as a free gift to the institution, to go towards maintaining free beds for the relief of the sick poor whose circumstances are such that they can give nothing whatever for the cost of their treatment. That is a condition of affairs which London should recognise, and try to immediately follow. The Hospital Saturday Fund authorities still adhere to the fatal principle of centralisation. The Government have called in the gentlemen who have been engaged in collecting these working-class contributions in the North of England and the Hospital Saturday Fund. The result of the action of those gentlemen in the North of England has shown that if you give the working man the opportunity he is proud to take up his proper position in regard to hospital work. What is the opinion of those gentlemen who are called in as experts? They say it is perfectly hopeless that you will get adequate support from the working classes in London until you decentralise. The present system is to require that all working men who take an interest in the Hospital Saturday Fund shall come to a central hospital in the centre of London, and that everything shall be done from that centre. So long as that system prevails, so long is it really impossible for the working classes as a whole to take up this question and to really contribute to the hospitals what they would desire to do on the provincial basis. What I would suggest is this: Let each large hospital set about this question of working-class contributions, and communicate in its own district with works situated in that district, and do exactly what they have done in the provinces. In the congregations there are ministers and churchwardens. In the workshop there are the master, the foreman, and the workmen, and permission must be got in the workshops, as you have to get the support of the clergy, to address the working men in the dinner hour, and enlist the sympathy of the foremen if you wish to increase the subscriptions and contributions of the working classes. I wish to show what is really the Pay System, but I will content myself by showing you that special hospitals have one great fault. Their promoters have said, "We must have money," and as a consequence they have wasted the voluntary contributions to the charity in raising a large sum. As much as £3,000 has been raised, and only £1,000 of that has gone to relieve sickness, because it has cost £2,000 to raise it. That is a great fault, but there is this much to be said in favour of special hospitals, that when their promoters saw that they had failed in this, they tried to meet it honestly by adopting the Pay System, and the Pay System, as so adopted, is admirable in this sense that it throws the proof of inability to pay upon each individual patient who seeks relief in the institution, and if they cannot show that they are unable to pay, they are expected to pay something; and I would ask anyone whom my words may reach, to take any special hospital you like—the Throat Hospital, the Hospital for Paralysis, or any other you choose—and go and see the patients in the out-wards and the patients in the out-wards of a general hospital, and you will find, if you examine the cases, that they are practically the same people, and that some results which are produced in certain hospitals are capable of being produced in general hospitals, where, subject to the exceptions which I have already alluded, it is possible to introduce some system of pay. I object to the registration fee pure and simple, because it is neither fish, flesh, nor fowl, nor good red herring. I absolutely object to it, and it cannot be advocated on any sound grounds, financial or philanthropic, and certainly not on the grounds of those who have been engaged in building up our great charitable institutions of this country. I feel that Mr. Burdett-Coutts has done an invaluable service to the hospital system of this country by his paper. It is not only a valuable paper, but the vast care and pains which the collection of his facts must have cost him must strike and fix the attention of all those engaged in hospital administration in this country. The paper is not only a valuable contribution to hospital literature, but a most valuable practical gift to every person who is engaged in the management of hospitals in the United Kingdom.

